# A New Treatment for Infected Wounds

**Published:** 1916-08-19

**Authors:** 


### A NEW TREATMENT FOR INFECTED WOUNDS.

### Bismuth and Iodoform Paste.

Professor Rutherford Morison, the well-known Newcastle surgeon, has communicated to the *Lancet* * particulars of a new treatment for infected and suppurating wounds, which marks, in his opinion, a great advance on anything he has yet tried. He hesitates to recommend the method at present for use in quite recently inflicted wounds at or near the Front with quite as much positiveness and assurance as for cases seen in England; but his expectation is that fresh wounds will respond as satisfactorily to the treatment as older ones do, provided that free drainage is allowed for the former.

### Bipp: A Chemical Antiseptic.

The essence of the method is chemical and antiseptic, not serologous or aseptic after the manner of Sir Almroth Wright and others. The steps of the treatment are perhaps best described in Professor Morison's own words: "Under an anæsthetic, usually open ether, cover the wound with gauze wrung out of 1 in 20 carbolic acid, and clean the skin and the surrounding area with the same lotion. Open the wound freely, and, if possible, sufficiently to permit of inspection of its cavity. In doing this special regard must be paid to nerve trunks and muscular branches of nerves, since the division of blood vessels, excepting the largest, and of the muscles themselves does little harm as compared with that of the disability following nerve damage. Cleanse the cavity with dry sterile gauze, mops, Volkmann's spoon, etc., and remove all foreign bodies. Mop the surrounding skin and the wound cavity with methylated spirit. Cotton-wool mops conveying the spirit are used for this purpose, and are introduced on forceps. Fill up the whole wound with paste, dress it with sterile gauze, and cover all with an absorbent pad, which is held in position by sticking-plaster and a bandage. This dressing requires no change for days or weeks if the patient is free from pain or constitutional disturbance. Should, however, discharge come through, the stained part must be soaked in spirit and a gauze dressing, wrung out of the same, applied as a further covering."

The paste in question, for which Professor Morison proposes the name of "Bipp," is made of: Bismuth subnitrate, one part by weight; iodoform, two parts by weight; liquid paraffin, enough to make a thick paste. So far he has seen only one case which suggested iodoform poisoning as a result of this treatment, and then the symptoms were very mild. He thinks it possible that the paste may stimulate osteogenesis; at least there have been cases of rapid union of fracture and other observations tending to support this idea.

### The Control of Suppuration.

The results described are exceedingly good. In more than forty cases treated on these lines there has been no need to change a dressing sooner than was intended. There has been, except in head wounds, no spread of septic infection, no cellulitis nor abscess formation, and no aggravation of constitutional disturbance after the operation. Relief of pain and fall of pulse-rate and of temperature when they had been abnormal has been the invariable rule. The application of these methods, the professor remarks, is not limited to war surgery: he has used them with equal success for septic infections in civil surgery. Certainly the claims set forth for this simple treatment are sufficiently definite to render it imperative for a thorough trial of it in all war hospitals. Details of many of the author's cases are given in his article, and they are certainly full of interest and of evidence as to the value of the method.

The claims made for the preliminary treatment of wounds followed by filling them with "Bipp" can be briefly summarised. Healing can be secured by first intention under infrequent dressings and without drainage other than that allowed through gaps left by interrupted sutures. Acute abscesses can be opened and cleaned and made to heal by first intention, without further pus formation. Compound fractures can be safely plated when "Bipp" is used. No bad results have followed the use of the method so far. It will be interesting to read the experiences of other surgeons with this treatment; it is to be hoped those who try it will report their cases fully, whether favourable or not.

* August 12, 1916, p. 268.

